# Defining steps in RAVE-catalyzed V-ATPase assembly using purified RAVE and V-ATPase subcomplexes

**DOI:** 10.1016/j.jbc.2021.100703

**Published:** 2021-04-22

**Authors:** Michael C. Jaskolka, Maureen Tarsio, Anne M. Smardon, Md. Murad Khan, Patricia M. Kane

**Affiliations:** Department of Biochemistry and Molecular Biology, SUNY Upstate Medical University, Syracuse, New York, USA

**Keywords:** vacuolar ATPase, vacuole, lysosomal acidification, yeast, proton pump, assembly, regulation, RAVE, BLI, biolayer interferometry, MBP, maltose-binding protein, MBP-C, MBP-tagged subunit C, RAVE, *R*egulator of the H+-*A*TPase of *V*acuoles and *E*ndosomes, SEC, size-exclusion chromatography, TBSE, Tris-buffered saline with EDTA, V-ATPase, vacuolar H+-ATPase, Vph1NT, N-terminal domain of V_o_ subunit Vph1, YEP, yeast extract–peptone medium without glucose, YEPD, yeast extract, peptone, 2% dextrose

## Abstract

The vacuolar H^+^-ATPase (V-ATPase) is a highly conserved proton pump responsible for the acidification of intracellular organelles in virtually all eukaryotic cells. V-ATPases are regulated by the rapid and reversible disassembly of the peripheral V_1_ domain from the integral membrane V_o_ domain, accompanied by release of the V_1_ C subunit from both domains. Efficient reassembly of V-ATPases requires the *R*egulator of the H^+^-*A*TPase of *V*acuoles and *E*ndosomes (RAVE) complex in yeast. Although a number of pairwise interactions between RAVE and V-ATPase subunits have been mapped, the low endogenous levels of the RAVE complex and lethality of constitutive *RAV1* overexpression have hindered biochemical characterization of the intact RAVE complex. We describe a novel inducible overexpression system that allows purification of native RAVE and RAVE–V_1_ complexes. Both purified RAVE and RAVE–V_1_ contain substoichiometric levels of subunit C. RAVE–V_1_ binds tightly to expressed subunit C *in vitro*, but binding of subunit C to RAVE alone is weak. Neither RAVE nor RAVE–V_1_ interacts with the N-terminal domain of V_o_ subunit Vph1 *in vitro*. RAVE–V_1_ complexes, like isolated V_1_, have no MgATPase activity, suggesting that RAVE cannot reverse V_1_ inhibition generated by rotation of subunit H and entrapment of MgADP that occur upon disassembly. However, purified RAVE can accelerate reassembly of V_1_ carrying a mutant subunit H incapable of inhibition with V_o_ complexes reconstituted into lipid nanodiscs, consistent with its catalytic activity *in vivo*. These results provide new insights into the possible order of events in V-ATPase reassembly and the roles of the RAVE complex in each event.

Intracellular organelles are tuned to distinct pH ranges that support organelle function and overall cellular homeostasis. Disruptions in pH balance are associated with a wide range of diseases, including cancer, metabolic acidosis, and neurodegeneration (1, 2, 3, 4). The vacuolar H^+^-ATPase (V-ATPase) is a highly conserved and ubiquitous ATP-driven proton pump that is responsible for organelle acidification in virtually all eukaryotic cells (5). V-ATPases are large and multisubunit complexes that are composed of two subcomplexes, V_1_ and V_o_. The peripheral V_1_ subcomplex contains sites for ATP hydrolysis, and the integral membrane V_o_ subcomplex contains the proton pore. In yeast, V_1_ is composed of eight subunits designated A–H, whereas V_o_ is composed of seven subunits designated a, c, c’, c”, d, e, and f (6, 7). V-ATPases of higher eukaryotes lack V_o_ subunit c’, and several subunits of V_1_ and V_o_ are encoded by multiple isoforms (8). In yeast, all V-ATPase subunits are encoded by single-copy *VMA* genes except the V_o_ a-subunit (9). Yeast V_o_ a-subunit isoforms Vph1 and Stv1 target the V-ATPase to the vacuole and Golgi, respectively and give their respective V-ATPases distinct regulatory properties (9, 10, 11).

V-ATPase activity is regulated through reversible disassembly of the V_1_ and V_o_ subcomplexes (12, 13). This process is conserved across species and occurs in response to numerous stimuli to fine-tune V-ATPase activity (14, 15, 16, 17, 18, 19). In yeast, acute glucose deprivation induces release of both subunit C and the rest of the V_1_ subcomplex from V_o_ (12, 20). Within free V_1_, subunit H undergoes a large conformation change that inhibits ATP hydrolysis in V_1_ and is hypothesized to serve as an energy conservation mechanism (6, 21). Disassembly also induces conformational changes within free V_o_ that effectively close the proton pore and prevent dissipation of the proton gradient (22).

Glucose readdition triggers rapid V-ATPase reassembly (12). Efficient delivery of subunit C and V_1_ to V_o_ and their functional reassembly requires the yeast *R*egulator of the H^+^-*A*TPase of *V*acuoles and *E*ndosomes (RAVE) complex (23, 24, 25). The RAVE complex is a V-ATPase specific assembly factor that is essential for both the biosynthetic assembly and glucose-dependent reassembly of V-ATPases containing the Vph1 isoform (26). RAVE is a heterotrimeric complex composed of Rav1, Rav2, and Skp1 subunits, with Rav2 and Skp1 interacting with the N-terminal and C-terminal portions of Rav1, respectively (27). Rav2 interacts with V_1_ subunit C, whereas Skp1 does not interact with any V-ATPase subunits (23, 25). Rav1 contains interaction sites for V_1_, V_1_ subunit C, and V_o_ (27), suggesting it may have a critical role in V-ATPase reassembly as well as serving as the central component of the RAVE complex. Recent structures of intact V-ATPases and V_1_ and V_o_ subcomplexes have highlighted the structural requirements for V-ATPase reassembly and suggested potential points of intervention by the RAVE complex. In the assembled V-ATPase, the N-terminal domain of V_o_ subunit Vph1 (Vph1NT) interacts with one of the three EG stalks and subunit C of V_1_ to form a high-avidity interaction that has been proposed as an important target in reassembly (28, 29). Interestingly, there are interaction sites for an EG stalk, Vph1NT, and subunit C in close proximity on Rav1 (27).

This could suggest that RAVE functions as a scaffold for this ternary interaction to occur, but binding affinities between these V-ATPase subunits have only been measured in the absence of RAVE, so the influence of RAVE on these interactions is unknown. In addition, the C-terminal domain of subunit H is rotated by 150° in the V_1_ complex, resulting in new contacts with V_1_ subunits and masking of the interaction between subunit H and Vph1NT of V_o_ (6, 28). This conformational change in subunit H is associated with entrapment of an inhibitory ADP in one of the catalytic sites and serves not only to inhibit ATPase activity in V_1_ but also to inhibit binding to V_o_ (28, 29). While there is no evidence for direct interaction of subunit H with RAVE, it is possible that conformational changes induced by RAVE binding to V_1_ and/or subunit C could permit release of inhibition and help position the H subunit for binding to Vph1NT. Finally, the nature of the glucose signal for reassembly is not understood. However, we recently determined that the RAVE complex can be recruited from the cytosol to the vacuolar membrane upon glucose readdition, even in the absence of binding to V_1_ or subunit C (27, 30). This indicates that the interaction between the RAVE complex and Vph1NT (as part of the V_o_ complex in the membrane) is sensitive to extracellular glucose levels.

*RAV1* and *RAV2* are expressed at much lower levels than V-ATPase subunits, and constitutive overexpression of *RAV1* is lethal (31). The limited endogenous levels of the Rav1 and Rav2 subunits have hindered efforts to purify sufficient RAVE complex for the type of *in vitro* binding and reassembly experiments that would allow us to fully characterize the catalytic role of RAVE in V-ATPase reassembly. Here, we describe a novel method to acutely overexpress Rav1 and Rav2 and purify enough RAVE and RAVE–V_1_ complexes for binding and reconstitution studies. Both RAVE alone and RAVE–V_1_ complexes copurify with substochiometric levels of subunit C. Purified RAVE–V_1_ complexes bind tightly to exogenously supplied subunit C, but subunit C binds weakly to RAVE without V_1_. RAVE–V_1_ complexes have no MgATPase activity, suggesting that subunit H remains in its inhibitory conformation in V_1_ while bound to RAVE. Neither RAVE nor RAVE–V_1_ binds Vph1NT *in vitro*. RAVE–V_1_ in combination with exogenous C subunit was unable to assemble with intact V_o_ complexes reconstituted into lipid nanodiscs. However, purified RAVE accelerates assembly of active and concanamycin-sensitive V-ATPases when combined with a V_1_ complex containing a mutant H subunit incapable of assuming inhibitory conformation (6, 32) along with subunit C and V_o_-containing nanodiscs. Taken together, these results suggest that RAVE–V_1_ is a stable intermediate complex that acquires subunit C in the process of V-ATPase reassembly *in vivo*. *In vitro*, RAVE cannot “unlock” V_1_ from the inhibited conformation generated by subunit H, but when this inhibition is relieved by mutation, RAVE is able to catalyze V-ATPase assembly. These results provide important insights into the role of RAVE in V-ATPase assembly.

## Results

### Overexpression of RAVE subunits

How RAVE primes V_1_ subcomplexes and subunit C for reassembly is unknown (30), and examining the interactions of the intact RAVE complex with V_1_ complexes and isolated subunit C is critical to answering this question. However, *RAV1* and *RAV2* are expressed at only ∼10% the levels of the V-ATPase subunits (33), and constitutive overexpression of Rav1 is lethal because it titrates Skp1 away from other essential subcomplexes (31). As a result, isolation of sufficient quantities of RAVE complex for biochemical characterization, quantitative binding studies, and reassembly assays have been difficult.

**Figure 1:**
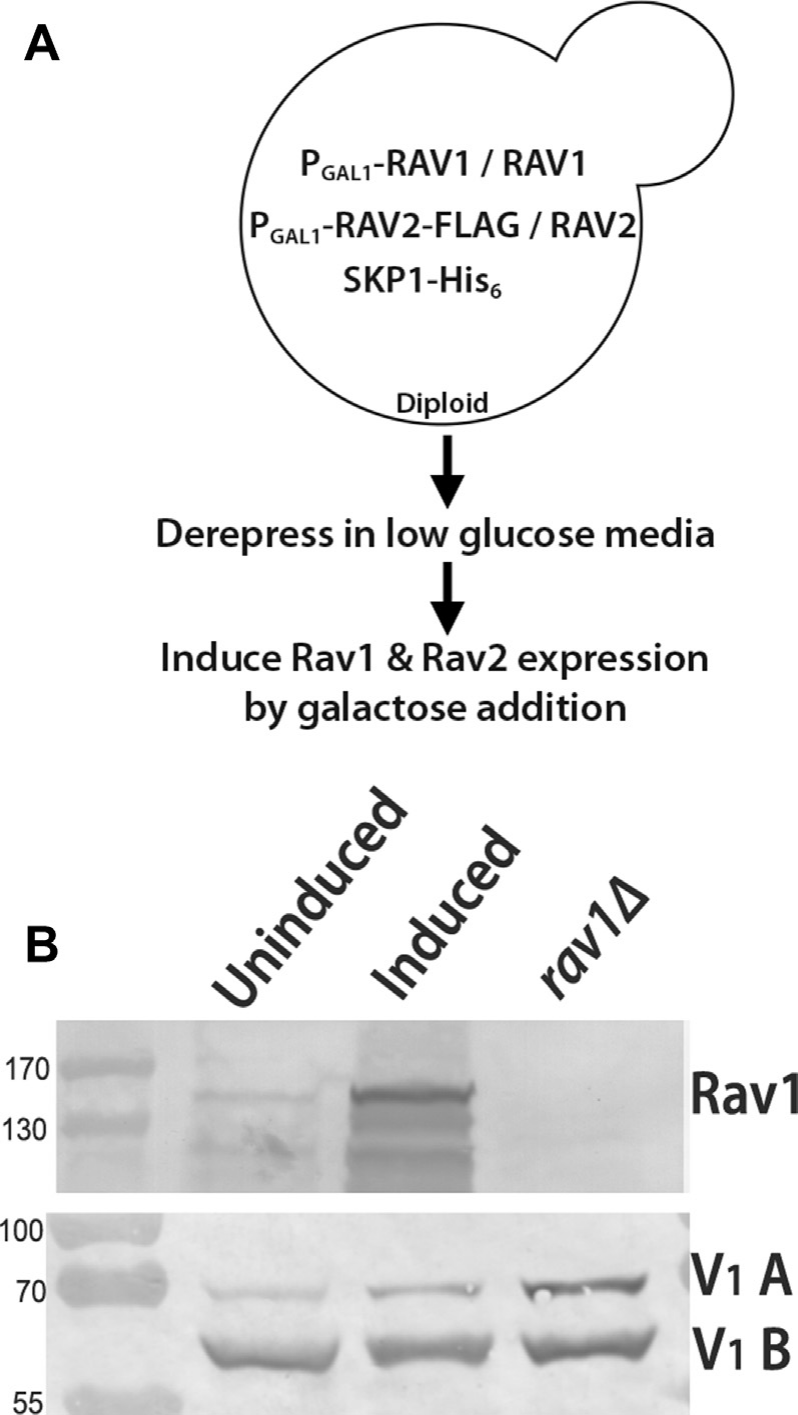
**An inducible system for overexpression of Rav1 and Rav2-FLAG.***A*, scheme for overexpression of *RAV1* and *RAV2*-FLAG. *B*, Western blot to analyze Rav1 expression before and after galactose induction. Cell lysates derived from equivalent numbers of cells from uninduced and induced diploids, as well as a *rav1Δ* strain, were subjected to SDS-PAGE and run on two separate gels. The *top* blot was probed with rabbit anti-Rav1 antibody, and the *bottom* blot was probed with mouse monoclonal antibodies against the A and B subunits of the V-ATPase. V-ATPase, vacuolar H+-ATPase.

**Figure 2:**
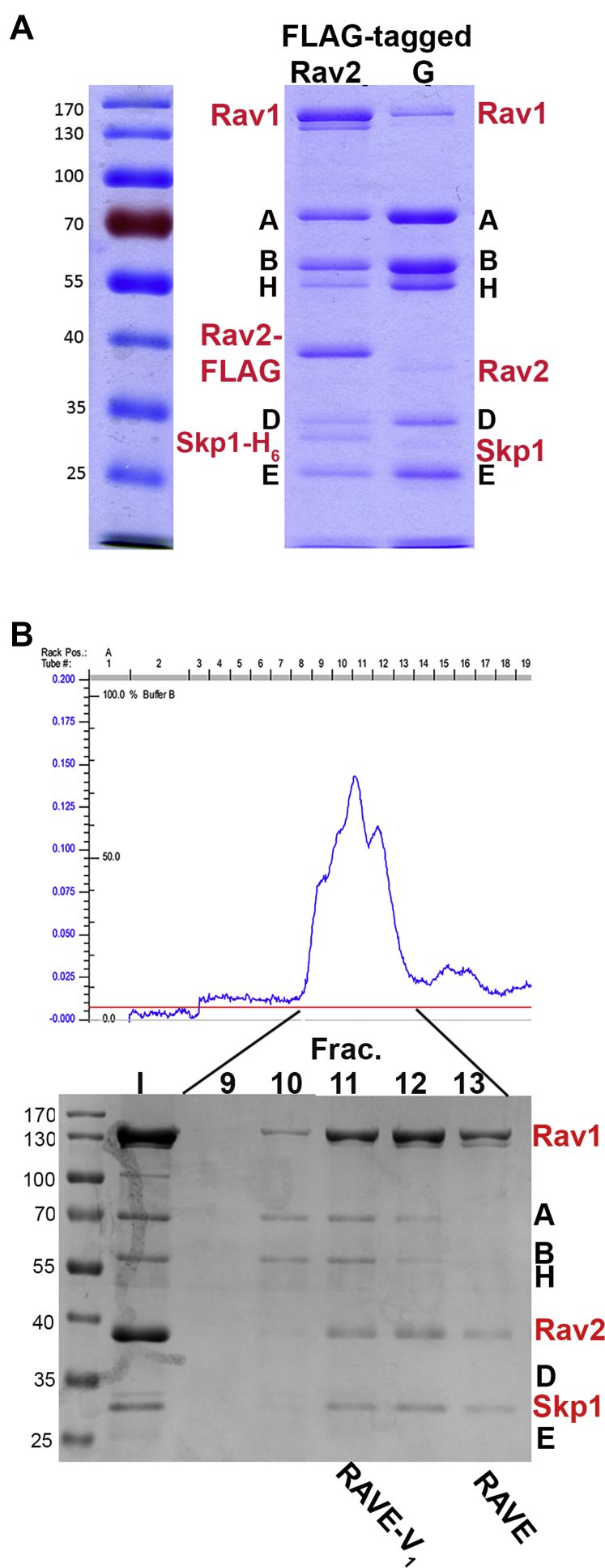
**Purification of RAVE–V**_**1**_**and RAVE complexes.***A*, affinity purification *via* FLAG-tagged Rav2. Coomassie-stained SDS-PAGE of a peak fraction from affinity purification with FLAG-tagged Rav2 is compared with a peak fraction of V_1_ affinity-purified *via* FLAG-tagged subunit G from a *vma5Δ* mutant. Positions of RAVE subunits are indicated in *red*, and V_1_ subunits are indicated in *black*. Samples are from a single gel, cut for better labeling. *B*, gel filtration profile for a sample affinity-purified *via* FLAG-tagged Rav2 as described in Experimental procedures section. Below the profile is an SDS-PAGE of the indicated fractions. RAVE–V_1_ is enriched in fraction 11, and RAVE is enriched in fraction 13. Fraction 12 appears to show a mixture of the two complexes. RAVE, *R*egulator of the H+-*A*TPase of *V*acuoles and *E*ndosomes.

To overcome these limitations, we designed a system for acute overexpression of *RAV1* and *RAV2* diagrammed in Figure 1. Briefly, the *RAV1* and *RAV2* genes were placed under control of the inducible yeast *GAL1* promoter (P_GAL1_) (34) in separate haploid cells of opposite mating type. Rav2 was tagged at the C terminus with a single copy of the FLAG (Sigma) epitope in the P_GAL1_–*RAV2* strain. Skp1 was epitope tagged with a hexahistidine tag in both haploid strains. The haploid strains were crossed, resulting in a heterozygous diploid with one copy of both *RAV1* and *RAV2* expressed from their native promoters and another copy of each subunit gene under the *GAL1* promoter (Fig. 1*A*). In order to overexpress *RAV1* and *RAV2*, yeast cells were grown in low glucose media until glucose exhaustion. Galactose was then added, and cells underwent one doubling before growth arrest. As shown in Figure 1*B*, this strategy yielded a significant increase in levels of the ∼150 kDa Rav1 protein. The Rav1 band is not present in a *rav1*Δ strain (Fig. 1*B*), but V_1_ subunits A and B are present in all three strains. Rav2-FLAG was undetectable in glucose-grown cells, as expected, but is expressed after galactose induction as shown in Figure 2.

### Purification of the RAVE and RAVE–V_1_ complexes

We purified RAVE complexes from the galactose-induced strain by affinity purification using the FLAG epitope on Rav2. As shown in Figure 2*A*, the other subunits of the RAVE complex, Rav1 and Skp1–His_6_, copurify with Rav2-FLAG, along with V_1_ subunits. In Figure 2*A*, the affinity-purified RAVE–V_1_ complexes are compared with V_1_ complexes isolated *via* a FLAG-tagged V_1_G subunit (35) purified from a strain lacking subunit C and expressing *RAV1* and *RAV2* from their endogenous promoters. V_1_ complexes isolated from this strain contain detectable, but substoichiometric, levels of the RAVE complex. These results suggest that overexpressed Rav1 and Rav2 are able to assemble into intact RAVE complexes and in addition, bind to intact V_1_ complexes.

We hypothesized that affinity purification using Rav2-FLAG might isolate both RAVE in complex with V_1_ (RAVE–V_1_) as well as RAVE complexes alone. RAVE (molecular mass 217 kDa) and V_1_ (∼600 kDa) are large complexes. We utilized size-exclusion chromatography (SEC) to separate RAVE–V_1_ from free RAVE. Although RAVE–V_1_ and RAVE peaks partially overlapped, SEC yielded fractions that appear to be enriched in RAVE–V_1_ or RAVE alone (Fig. 2*B*). The fractions most enriched for RAVE–V_1_ and RAVE alone were used in subsequent experiments.

### Binding of RAVE–V_1_ and RAVE complexes to subunit C *in vitro*

**Figure 3:**
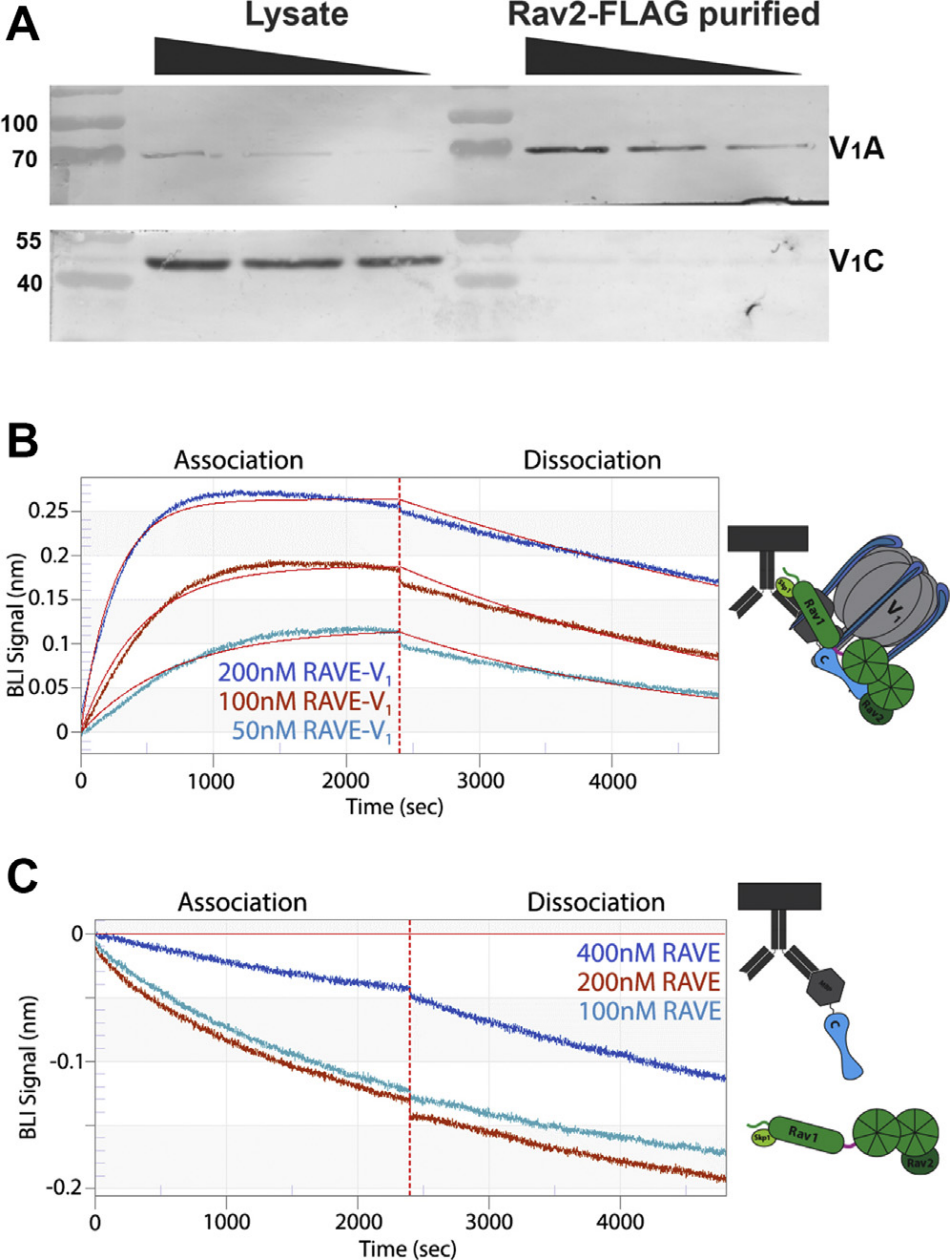
**Binding of subunit C to RAVE and RAVE–V**_**1**_**.***A*, limited copurification of subunit C with FLAG-tagged Rav2. Decreasing amounts of lysate from cells induced as described previously were loaded on the *left*, and decreasing amounts of a peak fraction from affinity purification *via* Rav2-FLAG were loaded on the *right*. After SDS-PAGE and transfer to nitrocellulose, the blot was cut and probed for V_1_ subunits A and B or subunit C. *B*, RAVE–V_1_ and RAVE purified as described in Figure 2*B* were tested for binding to MBP-tagged subunit C (MBP-C) by BLI. Anti-mouse IgG capturing biosensors were loaded with anti-MBP antibody, washed, and then bound to MBP-C. After further washing, biosensors were exposed to varied concentrations of RAVE–V_1_ (*B*) or RAVE alone (*C*) during the association phase. Sensors were then dipped into wells containing buffer alone, and dissociation was monitored. Background from RAVE–V_1_ and RAVE alone binding to sensors lacking MBP-C was subtracted for the analysis of kinetic data. The association and dissociation curves were fit to a global and single-site model. No *K*_*d*_ could be obtained for RAVE alone. The data are representative of three independent experiments. BLI, biolayer interferometry; RAVE, *R*egulator of the H+-*A*TPase of *V*acuoles and *E*ndosomes.

Previous results indicate that RAVE can bind to both cytosolic V_1_ complexes and to V_1_ subunit C (25, 27), but we noticed that subunit C appeared to be missing from the proteins copurified with Rav2-FLAG. In order to assess this more directly, we ran both the whole cell lysate and Rav2-FLAG affinity-purified proteins on SDS-PAGE and probed immunoblots for the relative levels of the V_1_ A and C subunits. As shown in Figure 3*A*, V_1_ subunit A is highly enriched by copurification with Rav2-FLAG, but subunit C is depleted from the FLAG-purified complexes. This suggests that subunit C does not copurify with Rav2-FLAG as well as the other subunits of the V_1_ complex. The lack of subunit C in the affinity-purified RAVE and RAVE–V_1_ complexes was surprising given that both RAVE and V_1_ contain multiple binding sites for subunit C, and subunit C coimmunoprecipitates with Rav1 (25, 27, 29, 36). To address this issue, we assessed whether RAVE–V_1_ was able to bind to subunit C *in vitro*. We utilized biolayer interferometry (BLI) to quantitate binding between bacterially expressed maltose-binding protein (MBP)–tagged subunit C (MBP-C) and purified RAVE–V_1_ in a strategy similar to that of the study by Sharma *et al.* (29, 32). Briefly, MBP-C was bound to BLI sensors loaded with anti-MBP antibody and then dipped into wells containing varied concentrations of RAVE–V_1_. Figure 3*B* shows that RAVE–V_1_ binds to subunit C in a concentration-dependent manner. Fitting these data, we obtained a *K*_*d*_ of 13.6 nM. Three independent RAVE–V_1_ preparations gave an average *K*_*d*_ of 18.1 ± 4.8 nM by BLI. Interestingly, this affinity is lower than the observed affinity of isolated V_1_ for subunit C (∼0.7 nM) but higher than the affinity of subunit C for isolated EG stalk complexes (∼42 nM) (32, 36, 37, 38). To address the contribution of the RAVE complex to the interaction between subunit C and RAVE–V_1_, we repeated the binding experiments with RAVE alone. When BLI sensors with bound MBP-C were dipped into wells containing the RAVE complex, we observed a slow decrease in BLI signal throughout the association and dissociation phases (Fig. 3*C*), suggesting little or no binding under these conditions.

**Figure 4:**
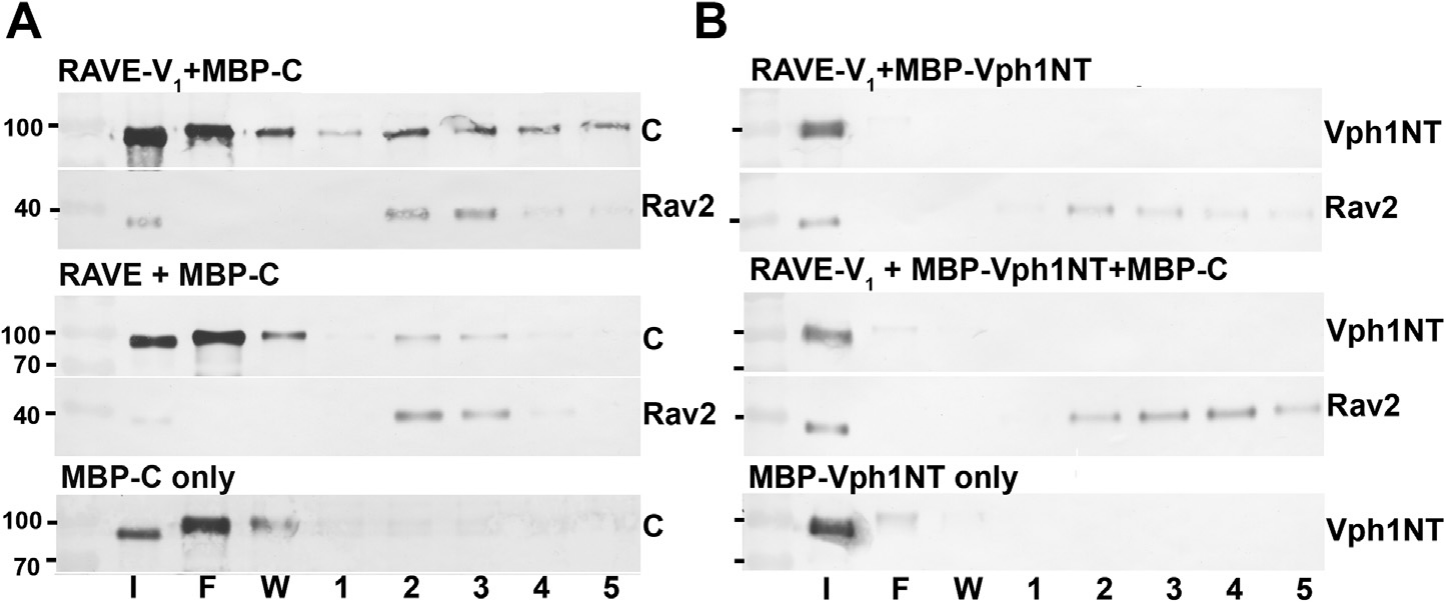
**Pulldowns of MBP-tagged subunit C and Vph1NT with RAVE and RAVE–V**_**1**_**.***A*, gel filtration fractions containing RAVE–V_1_ or RAVE were incubated with MBP-C and anti-FLAG M2 beads as indicated, then transferred to a column for elution. All blots were loaded with input (I), flowthrough (F), wash (W), and fractions collected after addition of FLAG peptide to elute bound protein. MBP-C was also incubated with beads in the absence of FLAG-tagged RAVE as a control. *B*, RAVE–V_1_ was incubated with MBP-Vph1NT as described in *A* in the presence and absence of MBP-C as indicated. MBP-Vph1NT with no added RAVE–V_1_ is included as a control. MBP, maltose-binding protein; MBP-C, MBP-tagged subunit C; RAVE, *R*egulator of the H^+^-*A*TPase of *V*acuoles and *E*ndosomes; Vph1NT, N-terminal domain of V_o_ subunit Vph1.

In order to assess binding by an independent method, we also bound RAVE–V_1_ and RAVE complexes purified by SEC to anti-FLAG beads and tested their ability to bind to purified MBP-C in pull-down experiments. As shown in Figure 4*A*, MBP-C bound and coeluted with RAVE–V_1_ complexes from the anti-FLAG resin, consistent with the tight binding observed by BLI. However, there was also some coelution of MBP-C with RAVE alone, suggesting weak binding that was not detected by BLI. MBP-C subunit did not bind to FLAG resin the absence of RAVE or RAVE–V_1_ (Fig. 4*A*, *bottom*). Taken together, these data indicate that RAVE–V_1_ binds to subunit C more tightly than RAVE alone but less tightly than V_1_ alone.

### Neither RAVE nor RAVE–V_1_ bind to Vph1NT *in vitro*

The interaction between Rav1 and Vph1NT is essential for recruitment of RAVE and V_1_ to the vacuolar membrane (30). Given the importance of this interaction for the function of RAVE, we assessed the binding of RAVE–V_1_ and RAVE to purified Vph1NT. Binding of RAVE and RAVE–V_1_ to purified MBP-tagged Vph1NT (MBP-Vph1NT) was tested by BLI as described previously for MBP-C. Interestingly, although both RAVE and V_1_ contain binding sites for Vph1NT (27, 39), we observed only a slow dissociation from the BLI sensors during the association phase that continued throughout the dissociation phase, suggesting no binding under these conditions (data not shown). We also tested for binding of MBP-Vph1NT to RAVE–V_1_ bound to anti-FLAG beads in pull-down experiments (Fig. 4*B*), but no binding was observed. Similar results were seen when MBP-Vph1NT was incubated with RAVE alone (data not shown). We reasoned that addition of subunit C might improve binding of RAVE–V_1_ to Vph1NT and repeated the experiment with both expressed MBP-C and MBP-Vph1NT present (Fig. 4*B*). However, there was still no binding of Vph1NT to FLAG-tagged RAVE–V_1_ complexes. These data suggest that neither RAVE nor RAVE–V_1_ bind to Vph1NT under these conditions, even in the presence of added subunit C.

### Can RAVE catalyze V-ATPase reassembly *in vitro*?

Wildtype V_1_ complexes do not readily bind to V_o_ complexes *in vitro*, even in the presence of added subunit C (6, 29, 32, 38). This has been attributed to “locking” of V_1_ by rotation of subunit H into an inhibitory conformation. In support of this, Vph1NT binds to the isolated H subunit *in vitro* but not to V_1_ complexes containing subunit H (28). The yeast V_1_ structure revealed a 150° rotation of the C-terminal domain of subunit H that brought an inhibitory loop into proximity with V_1_ subunits B and D (6). Subunit H also silences the MgATPase activity of V_1_ complexes (21). In order to determine whether binding to RAVE releases subunit H from the inhibitory conformation in V_1_, we first measured the MgATPase activity of purified RAVE–V_1_. However, like wildtype V_1_ complexes, RAVE–V_1_ complexes have no MgATPase activity. This indicates V_1_ remains locked, with the H subunit in its inhibitory conformation, when it is in complex with RAVE. The failure of RAVE–V_1_ to bind to Vph1NT is consistent with these data.

The function of the RAVE complex is to catalyze reassembly of cytosolic V_1_ complexes and subunit C with V_o_ complexes at the vacuolar membrane. We initially asked whether RAVE could catalyze V-ATPase reassembly *in vitro* by combining purified RAVE–V_1_, purified subunit C, and Vph1-containing V_o_ subcomplexes reconstituted into nanodiscs (32, 40). We tested for functional reconstitution by measuring acquisition of concanamycin-sensitive ATPase activity. Concanamycin A binds in the V_o_ sector of V-ATPases and thus can only inhibit MgATPase activity in fully assembled complexes (41). However, no measurable MgATPase activity, even in the absence of concanamycin A, was observed when RAVE–V_1_ was combined with subunit C and V_o_ nanodiscs.

**Figure 5:**
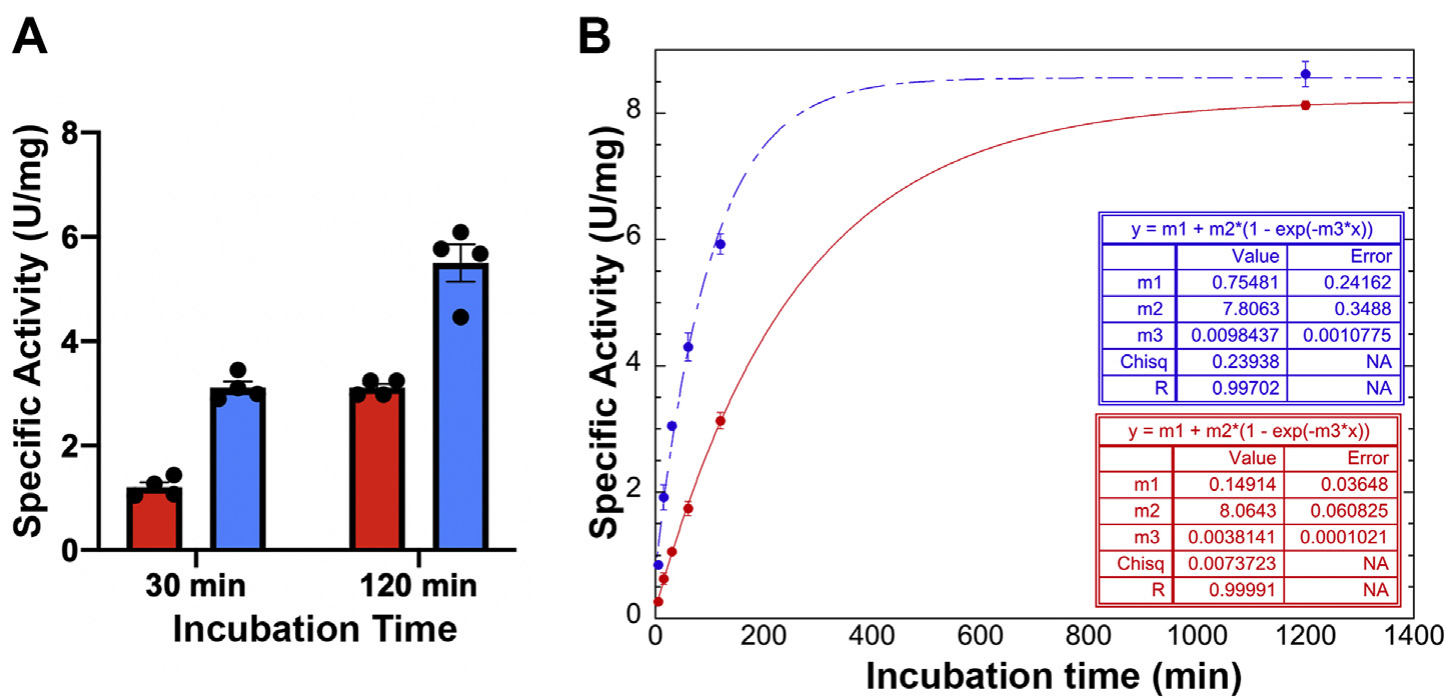
**Reconstitution of concanamycin-sensitive V-ATPase complexes in the presence and absence of RAVE.***A*, RAVE enhances V-ATPase reassembly and acquisition of concanamycin A–sensitive activity. Activity for two replicates from each of two independent experiments (using different preparations of components) is plotted for two time points of incubation. *Red columns*, no RAVE added; *blue columns*, RAVE added. Error bars represent SEM. *B*, time course of V-ATPase assembly. V_1_-H_chim_, Vph1–V_o_ nanodiscs and expressed subunit C were combined in a 1:1:3 M ratio and incubated at room temperature for the indicated times. Samples with RAVE had an equimolar concentration of RAVE, for a final of 210 nM each of RAVE, V_1_-H_chim_ and V_o_, and 630 nM subunit C, specific activity represents the ATPase activity sensitive to 200 nM concanamycin A. Each point represents the average of two replicates. Fits of each curve to a single exponential equation are indicated. RAVE, *R*egulator of the H^+^-*A*TPase of *V*acuoles and *E*ndosomes; V-ATPase, vacuolar H^+^-ATPase.

In previous experiments without RAVE, *in vitro* reassembly of V-ATPases was achieved, but only in the presence of a mutated subunit H lacking the inhibitory loop (H_chim_) (32). Under these conditions, reassembly was relatively slow compared with *in vivo* reassembly. Because our isolated RAVE–V_1_ was incapable of reassembly, we asked whether purified RAVE complexes might accelerate assembly of V_1_ containing H_chim_ with V_o_ nanodiscs. As described previously (32), V_1_ lacking subunit H was reconstituted with H_chim_ and combined with purified subunit C, and V_o_ nanodiscs, in the presence and absence of RAVE. Concanamycin-sensitive ATPase activity was assessed at various times. Figure 5*A* compares the increase in concanamycin-sensitive V-ATPase activity after 30 and 120 min of incubation in the presence and absence of RAVE. Acquisition of concanamycin-sensitive ATPase activity is significantly faster in the presence of RAVE. Importantly, mixtures of RAVE and V_1_-H_chim_ in the absence of either the subunit C or the V_o_ nanodiscs produced no detectable concanamycin-sensitive ATPase activity. Figure 5*B* shows a time course for acquisition of concanamycin A-sensitive activity in the presence and absence of RAVE. The data in the presence and absence of RAVE are both fit well to a single exponential curve (Fig. 5*B*). This suggests that there is a single rate-limiting step in the *in vitro* reassembly assay that is accelerated by the RAVE complex. The rate constant (m3) in the presence of RAVE is approximately 2.5 times that in the absence of RAVE. In addition, after 20 h of incubation, the mixtures with and without RAVE reach a similar activity suggesting that RAVE accelerates assembly of active V-ATPase complexes rather than assembling more complexes. These results indicate that purified RAVE is competent to catalyze V-ATPase assembly but only if the inhibition of V_1_ by subunit H is reversed.

## Discussion

These experiments provide important insights into both the order of events in RAVE-dependent V-ATPase reassembly and the roles of RAVE during reassembly, as well as indicating what the RAVE complex alone cannot do. Isolation of V_1_ complexes with FLAG-tagged Rav2 supports the tight interaction of cytosolic RAVE with V_1_ that was previously indicated by coimmunoprecipitation of the two complexes from glucose-deprived cells (23, 24). In contrast, the low level of subunit C isolated with FLAG-tagged Rav2 was somewhat surprising given the evidence of multiple binding sites for subunit C in both RAVE and V_1_. This is the first of several pieces of evidence indicating that a complex series of conformational changes may occur during RAVE-induced V-ATPase assembly. Previous data indicated that subunit C is copurified at low levels with cytosolic V_1_ under glucose deprivation conditions (42), but *in vitro*, purified subunit C binds to purified V_1_ with a high affinity (0.7 nM (32)). One explanation for this could be that *in vivo*, RAVE sequesters subunit C after release from the membrane, but the data presented here make that explanation unlikely. Interestingly, the affinity of purified RAVE–V_1_ for subunit C measured in Figure 3 (13.6 nM) is lower than the affinity of subunit C for V_1_ alone but slightly higher than the affinity of subunit C for an isolated EG heterodimer (42 nM (37)). Since both RAVE and subunit C bind to EG stalks of V_1_, the RAVE–V_1_ interaction could place V_1_ into a conformation that decreases affinity between V_1_ and subunit C. Alternatively, RAVE binding to V_1_ could place RAVE in a conformation that exposes its binding sites for subunit C. The affinity between RAVE–V_1_ and subunit C could be due to multiple interactions that would result in a high-avidity interaction between the three, as is seen between EG2–aNT–C_foot_ (38, 39). More data will be required to fully parse out the contributions of the different binding sites, but the data are consistent with the RAVE–V_1_ complex acting as an early intermediate in V-ATPase reassembly that acquires subunit C during a subsequent step. *In vivo*, this step could require release of subunit C from an inhibitory interaction or conformation in the cytosol. For example, subunit C has been shown to contain binding sites for the cytoskeleton (43, 44) that could help determine its availability for other interactions.

It is also significant that V_1_ appears to remain in its subunit H–inhibited conformation, incapable of MgATP hydrolysis, when in complex with RAVE. This is consistent with RAVE–V_1_ as a stable intermediate when cells are deprived of glucose and ATP levels are low (20). One could also have envisioned release of V_1_ from H subunit inhibition as a function of RAVE during V-ATPase reassembly. However, our data indicate that RAVE cannot reverse inhibition of ATPase activity because RAVE–V_1_ remains in its inhibited conformation. RAVE–V_1_ neither binds to Vph1NT (Fig. 4) nor assembles with V_o_ complexes in nanodiscs. In contrast, when inhibition by subunit H was relieved by mutation, RAVE was able to promote assembly of a functional V-ATPase from V_1_, subunit C, and V_o_ nanodiscs. These results suggest that release of H subunit inhibition, which also appears to release trapped MgADP (6), must be catalyzed by factors other than RAVE *in vivo*. It is unlikely that increased ATP levels resulting from glucose restoration to glucose-deprived cells are sufficient, because we previously showed that yeast cells acutely deprived of glucose show an initial drop in ATP levels that recovers quickly, even before glucose readdition (20). In addition, although ATP hydrolysis is required for V-ATPase disassembly and may play a role in reassembly (20, 29), we found that neither ATP nor a nonhydrolyzable analog influenced the RAVE–V_1_ interaction in BLI experiments (data not shown).

Interestingly, although we identified a binding site for Vph1NT in the center of the Rav1 subunit and demonstrated direct binding to a Rav1 fragment containing amino acids 679 to 898 *in vitro* (30), we also detected no binding of the purified RAVE to Vph1NT *in vitro*. The experiments described here use Vph1NT with an N-terminal MBP tag, and it is possible that the tag could compromise binding. However, in two-hybrid assays, N-terminally tagged Vph1NT interacted strongly with both intact Rav1 and the Rav1 (679–898) fragment (27). The lack of Vph1NT binding to RAVE complexes makes sense in the context of the physiological role of RAVE in reassembly, in which RAVE should only bind to V_o_ when delivering V_1_ and subunit C. These results suggest that the binding site for Vph1NT must be hidden in the intact RAVE complex.

RAVE-catalyzed V-ATPase reassembly is dependent on glucose signaling in yeast (30). Although the exact signals and pathways remain unclear, our data suggest at least two potential points in reassembly that might be impacted by cellular signals. First, the H subunit–mediated inhibitory conformation in V_1_ must be reversed for reassembly to occur, and it is clear that RAVE binding alone is insufficient to reverse this conformation. H subunit inhibition could potentially be released before or after binding to subunit C but is likely to be coordinated with binding to V_o_ at the vacuolar membrane to prevent ATP hydrolysis that is uncoupled from proton transport. Second, we recently showed that recruitment of the RAVE complex to the vacuolar membrane is glucose dependent and can occur in the absence of binding to subunit C or V_1_ (27, 30). These results indicate that the Rav1–Vph1NT interaction is a key glucose-sensitive interaction required for V-ATPase reassembly that could be a second point of intervention by cellular signals during *in vivo* reassembly (30). Although RAVE did promote assembly *in vitro* in Figure 5, RAVE concentrations relative to V_1_ and V_o_ are higher in this experiment than *in vivo*, and assembly was still slower than in cells, suggesting signaling may accelerate RAVE recruitment to the vacuolar membrane in cells.

**Figure 6:**
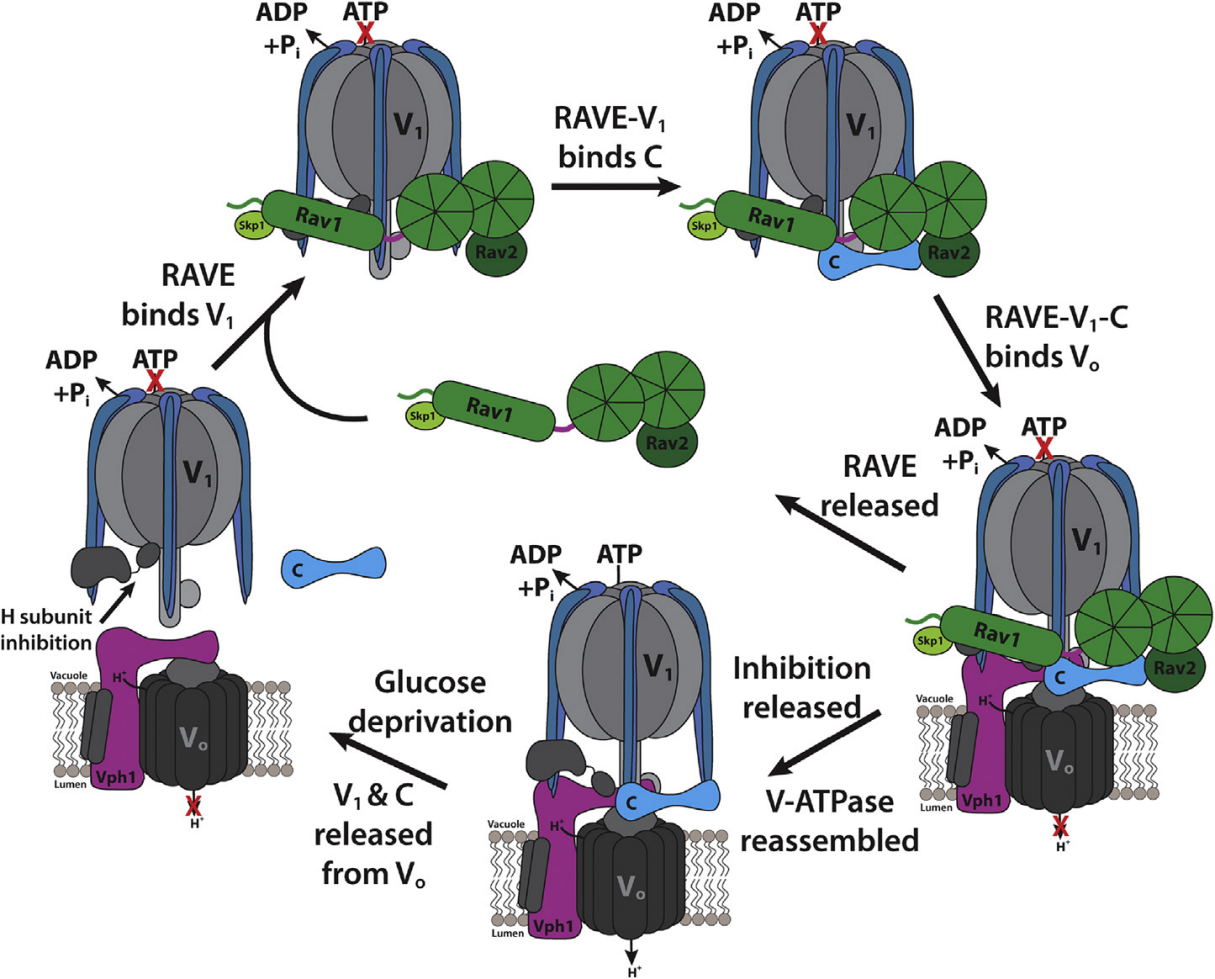
**Model for RAVE-driven catalysis of V-ATPase reassembly.** RAVE, *R*egulator of the H^+^-*A*TPase of *V*acuoles and *E*ndosomes; V-ATPase, vacuolar H^+^-ATPase.

Taken together, our results indicate that RAVE–V_1_ is the first intermediate in the reassembly pathway. RAVE–V_1_ complexes are competent to bind to subunit C as it becomes available. RAVE is then likely to have a key role in directing bound V_1_ and subunit C to V_o_ at the vacuolar membrane and may act as a scaffold in re-establishment of V_1_–V_o_ interactions, as suggested previously. The final step of binding of V_1_ and subunit C to V_o_ may be the rate-limiting step that is accelerated by RAVE. Specifically, RAVE may help to align V_1_, subunit C, and V_o_ for optimal assembly and thus accelerate their association and coupling. The proposed steps in the RAVE-driven assembly cycle are shown in Figure 6. *In vivo*, multiple cycles of binding, recruitment to the vacuolar membrane, reassembly, and RAVE release are likely to occur, given that RAVE subunits are present at much lower endogenous levels than V-ATPase subunits.

## Experimental procedures

### Materials and growth media

Oligonucleotides were purchased from MWG Operon. Anti-FLAG-M2 resin, mouse anti-FLAG-M2 antibody, and FLAG peptide were purchased from Sigma. Amylose resin was purchased from New England Biolabs. Concanamycin A was purchased from Santa Cruz Biotechnology. Media for growth of yeast and *Escherichia coli* were purchased from Fisher Scientific. Yeast were maintained in yeast extract, peptone, 2% dextrose (YEPD) medium buffered to pH 5 with 50 mM potassium phosphate and 50 mM potassium succinate. Antibiotic selections in yeast were performed in YEPD containing 200 μg/ml G418 (Thermo Fisher Scientific), 100 μg/ml nourseothricin (Jena Bioscience), or 200 μg/ml hygromycin B (Invitrogen).

### Generation of yeast strains

RAVE subunits were tagged, and their promoters replaced by an established PCR-based strategy in which the desired tag and selectable marker were amplified with oligos that also contained approximately 50 bp of homology immediately upstream and downstream of the position of tag insertion (45, 46, 47). A C-terminal His_6_ tag was inserted into Skp1 by amplifying a 6xGly–6xHis–*KanMX6* cassette from the pFA6a–6xGly–His-tag–*KanMX6* plasmid obtained from Addgene (47). The PCR product was transformed (48) into haploid yeast strains SF838-5Aα and SF838-5Aa, transformants were selected on YEPD containing G418, and insertion of the tag was confirmed by PCR and sequencing. The *KanMX6* marker was then replaced with the hygromycin resistance gene (*HphMX4*) from pBS35 (University of Washington Yeast Resource Center) using a similar strategy, and transformants were selected on YEPD containing hygromycin. The *GAL1* promoter was added upstream of *RAV1* and *RAV2* in the Skp1-tagged strains by amplifying a cassette containing the *kanMX6* marker upstream of the *GAL1* promoter from the pFA6a–*kanMX6*–P_GAL1_ plasmid (46), then transforming the PCR product into the Skp1–His_6_-containing SF838-5Aα and SF838-5Aa strains, respectively, and selecting transformants on YEPD containing G418. In the SF838-5Aa strain, the *kanMX6* marker upstream of *P*_*GAL1*_*-RAV2* was then switched to *natMX4* as described (49), and *RAV2* was C-terminally tagged with FLAG by amplifying the FLAG tag in combination with the *kanMX6* marker from plasmid pFA6a–6xGly–FLAG:KanMX6 (47) from Addgene as described previously. The genotypes of the final two haploids are SF838-5Aα P_GAL1_-RAV1 (*MATα kanMX6:P*_*GAL1*_*-RAV1 SKP1-6xGly-6xHis-hphMX4 ura3-52 leu2-3,112 his4*) and SF838-5Aa P_GAL1_-Rav2-FLAG (*MATa natMX4:P_GAL1_-RAV2-FLAG SKP1-6xGly-6xHis-hphMX4 ura3-52 leu2-3,112 his4*). These two strains were crossed, and zygotes were isolated under a dissecting microscope. The genetic markers of the resulting diploid were confirmed.

### Overexpression of Rav1 and Rav2-FLAG

Overexpression of the RAVE subunits was achieved by first inoculating 50 ml of YEPD with the diploid strain described previously, then growing the cells to log phase. A volume of cells equivalent to 50 at absorbance at 600 nm units was pelleted and resuspended in 3 ml yeast extract–peptone medium without glucose (YEP), then added to 1 l YEP media containing 0.2% (w/v) glucose that had been adjusted to pH 5 with HCl. Cells were shaken at 30 °C, grown to a density of ∼1 at an absorbance at 600 nm/ml, and then galactose was added to a final concentration of 2% (w/v) to induce Rav1 and Rav2-FLAG overexpression. Growth was continued to a density of ∼2 at an absorbance at 600 nm/ml (∼16–18 h), then cells were pelleted, and the pellets were resuspended in 3 ml YEP. The cell suspension was transferred to a 50 ml conical tube, cells were pelleted again, and cell pellets were frozen at −80 °C. Samples of cells were removed before and after galactose induction to assess Rav1 overexpression.

### Purification of RAVE and RAVE–V_1_

Chilled PBS (137 mM NaCl, 2.7 mM KCl, 10 mM Na_2_HPO_4_, and 1.8 mM KH_2_PO_4_) or Tris-buffered saline with EDTA (TBSE) (50 mM Tris–HCl, 150 mM NaCl, and 2 mM EDTA) was adjusted to a pH of 7.4 with either HCl or NaOH. Purifications from both buffers gave very similar results, although there was some evidence that RAVE was more stable in TBSE. Yeast pellets were placed in an ice bath, brought to 35 ml with cold buffer, and protease inhibitors (final concentrations: 1 mM phenylmethylsulfonyl fluoride, 1 μg/ml pepstatin A, 1 μg/ml leupeptin, and 5 μg/ml aprotinin) were added. After thawing, cells were pooled and lysed with a microfluidizer. Cells were passed through the microfluidizer four times, placed on ice for 5 min, and the process repeated 5 to 6 times. After five repeats, lysis was checked microscopically; 20 passes through the microfluidizer typically gave at least 90% lysis. The lysate was spun down at 17,000 rpm in a Beckman JA20 rotor for 45 min at 4 °C, and the supernatant was decanted. The supernatant was then centrifuged at 60,000 rpm in a Beckman Ti70 rotor for 1.5 h. Additional protease inhibitors were added to the supernatant, which was then passed through a 0.45 μM filter. The lysate was gravity fed through a 5 ml anti-FLAG M2 affinity column twice. The column was washed with 25 ml buffer, and then the bound protein was eluted into 500 μl fractions after addition of 10 ml 180 μg/ml FLAG peptide. After elution, 5 μM DTT was added to each fraction. In order to identify peak fractions for further purification, 30 μl was removed from each fraction, diluted 1:1 with cracking buffer (50 mM Tris–HCl, pH 6.8, 8 M urea, 5% SDS, and 5% β-mercaptoethanol), and then heated at 95 °C for 10 min. About 30 μl of each diluted fraction was loaded for SDS-PAGE, and the resulting gels were stained with Coomassie blue.

For further purification by SEC, 2 ml of FLAG-purified proteins were loaded on a BioRad DuoLogic FPLC and separated on a Superdex 200 Increase 10/300 GL size exclusion column (GE Healthcare Life Sciences). About 0.5 to 1 ml fractions were collected, and peak fractions were identified by absorbance at 280 nm. About 30 μl from each fraction was prepared and run on SDS-PAGE as aforementioned to identify RAVE–V_1_ and RAVE-enriched fractions. Final protein concentrations were determined at 280 nm on a NanoDrop 2500 spectrophotometer.

### Expression and purification of bacterial constructs

MBP-Vph1NT (1–372) and MBP-C were expressed as previously described (37). Briefly, plasmids containing MBP-Vph1NT and MBP-C were transformed into BL-21 and Rosetta2 cells, respectively, for expression (28, 37). BL-21 and Rosetta cells were grown in Luria broth supplemented with 2% glucose with ampicillin (100 μg/ml) alone or in combination with chloramphenicol (34 μg/ml), respectively. Protein expression was induced by addition of 0.5 mM IPTG when cells reached an absorbance of 0.5 to 0.6 at 600 nm. Growth was continued for 16 h at 18 °C for MBP-Vph1NT and for 6 h at 30 °C for MBP-C. After induction, cells were pelleted and frozen at −80 °C.

MBP-tagged proteins were affinity purified on amylose columns, and peak fractions were collected, as described (27), except that after cell lysis, the supernatant was then applied to a 4 ml amylose column, the column was washed with 20 ml amylose column buffer, and then proteins were eluted with 10 mM maltose in amylose column buffer into 1 ml fractions. MBP was not cleaved from the tagged proteins, except in the case of the C subunit used in Figure 5, which was prepared as in the study by Oot *et al.* (37).

### BLI

All BLI experiments were performed on an Octet RED384 System in either PBS or TBSE (depending on the buffer used to obtain RAVE–V_1_ and RAVE for that experiment). Bovine serum albumin was added to a final concentration of 0.5 mg/ml to the buffer and all other components. MBP-C or MBP-Vph1NT was diluted to 5 μg/ml, and anti-MBP antibody was diluted to 1 μg/ml in buffer. About 200 μl of sample was added to each well in a 96-well BLI plate, loaded into an Octet-RED system, and maintained at 22 °C. Each biosensor was stirred at 1000 rpm and a rate of 5 s^−1^. Anti-mouse IgG-capturing biosensors (FortéBio, AMC biosensors catalog no.: 18-5088) were prewetted and equilibrated in buffer, then loaded with anti-MBP antibody. The sensors were washed to reduce nonspecific binding, then dipped into wells containing 5 μg/ml MBP-tagged protein for experimental wells and buffer for reference wells and washed again. Sensors were then dipped into wells containing RAVE, RAVE–V_1_, or buffer (as a control) for 2400 s for the association phase, then transferred to buffer for 2400 s for the dissociation phase. For data analysis, background-subtracted data were obtained by subtracting the reference wells (no MBP-C or MBP-Vph1NT) from experimental wells. The association and dissociation curves were fit to a global single-site binding equation to obtain the *K*_*d*_.

### Pulldowns of MBP-tagged subunits with RAVE and RAVE–V_1_

Gel filtration fractions containing RAVE–V_1_ or RAVE were combined with a 1.5-fold excess of purified MBP-Vph1NT or MBP-C in PBS containing 0.5 mg/ml bovine serum albumin and rocked for 1.5 h at 4 °C with 200 μl of anti-FLAG M2 beads that had previously been washed with the same buffer. The mixtures were transferred to a small column, washed with buffer, and the bound protein was eluted with FLAG peptide as described previously. Input samples and fractions were precipitated with 15% trichloroacetic acid, and the pellets were solubilized in cracking buffer. All samples were separated by SDS-PAGE and transferred to nitrocellulose. The resulting Western blots were probed with mouse anti-FLAG (to detect Rav2-FLAG), rabbit anti-Vma5 (to detect subunit C), and mouse 10D7 monoclonal antibody (to detect Vph1NT), followed by alkaline phosphatase–conjugated goat antimouse or anti-rabbit antibody. The rabbit anti-Vma5 polyclonal antibody was a generous gift from Tom Stevens.

### Functional reconstitution of the V-ATPase *in vitro*

V_1_ containing H_chim_, Vph1-containing V_o_ nanodiscs, and subunit C were purified and reconstituted into functional V-ATPase as described by Stam and Wilkens (50) and Sharma *et al.* (32). The three components were mixed in 1:1:3 M ratio, respectively, and incubated at room temperature for the indicated times. In RAVE-containing mixtures, RAVE was added at an equimolar ratio to V_1_H_chim_ and V_o_. At the indicated times, samples were withdrawn to measure the concanamycin A–sensitive ATPase activity at 37 °C using ATP-regenerating system as described by Oot *et al.* (6). Briefly, 1 ml of ATPase assay (50 mM Hepes, 25 mM KCl, 0.5 mM NADH, 2 mM phosphoenolpyruvate, 5 mM ATP, 30 units each of lactate dehydrogenase and pyruvate kinase, and pH 7.5) was prewarmed to 37 °C and supplemented with 4 mM MgCl_2_. About 5 to 10 μg of sample was added, and the decrease in absorbance at 340 nm was measured using a temperature-controlled Varian CARY100 Bio UV-Visible Spectrophotometer in kinetics mode. About 200 nM of concanamycin A was added into the assay after establishment of the initial rate in order to assess sensitivity. Concanamycin A–sensitive V-ATPase activity is expressed as units/milligram where a 1 unit = 1 μmol ATP hydrolyzed/min.

## Data availability

All strains and plasmids, as well as oligonucleotide sequences used in strain construction, are available upon request from Patricia Kane, SUNY Upstate Medical University, kanepm@upstate.edu.

## Conflict of interest

The authors declare that they have no conflicts of interest with the contents of this article.
